# alpha1-Antitrypsin deficiency and hepatocellular cancer.

**DOI:** 10.1038/bjc.1978.93

**Published:** 1978-04

**Authors:** M. C. Kew, R. Turnbull, I. Prinsloo


*Br. J. Cancer* (1978) **37**, 635

***Short Communication***

### $\alpha_1$-ANTITRYPSIN DEFICIENCY AND HEPATOCELLULAR CANCER

M. C. KEW*, R. TURNBULL† AND I. PRINSLOO†

*From *the Department of Medicine, University of the Witwatersrand and Johannesburg Hospital, the South African Primary Liver Cancer Research Unit, and †Department of Microbiology of the National Research Institute for Occupational Diseases of the South African Medical Research Council, Johannesburg, South Africa*

Received 15 November 1977 Accepted 29 December 1977

$\alpha_1$-ANTITRYPSIN (AAT), a glycoprotein synthesized in the liver, is responsible for about 90% of the inhibitory capacity of human serum for trypsin, and also for the inhibition of other proteolytic enzymes such as elastase, collagenase and leucocyte proteases. Synthesis of AAT is controlled by a pair of genes at one locus for which as many as 24 alleles have been described. These alleles are inherited in an autosomal codominant manner. They are responsible for the different structural variants of the AAT molecule, which can be detected by their different electrophoretic mobilities. The Z, or slowest variant‡, in either its homozygous or heterozygous form, may be the cause of a deficiency of AAT in the serum.

AAT deficiency has been shown to be associated with a variety of diseases, but particularly with chronic obstructive pulmonary disease (Ganrot *et al.*, 1967) and liver disease (Berg and Eriksson, 1972; Feldman *et al.*, 1974; Sharp, 1976). The latter takes the form of cholestatic jaundice in infancy (neonatal hepatitis), childhood cirrhosis and, less often, cirrhosis in adults. In addition, a few cases of hepatocellular (HCC) or cholangiocellular cancer have been described in patients with homozygous (ZZ) or heterozygous (MZ) AAT deficiency (Ganrot *et al.*, 1967; Berg and Eriksson, 1972; Eriksson and Hagerstrand, 1974; Aagenaes *et al.*, 1974; Lieberman, 1974; Rawlings, *et al.*, 1974; Williams and Fajardo, 1974; Lieberman *et al.*, 1975; Zwi *et al.*, 1975). A characteristic finding in individuals with both homozygous or heterozygous AAT deficiency, in the presence or absence of liver disease, is the accumulation of periodic acid–Schiff (PAS)-positive, diastase-resistant globules in periportal hepatocytes (Sharp, 1971). These globules consist of aggregates of an asialo-antitrypsin within the dilated endoplasmic reticulum (Eriksson and Larsson, 1975).

The frequency with which AAT deficiency predisposes to HCC, if indeed it does so, is not known. In 2 retrospective studies (Norkin and Campagna-Pinto, 1968; Berg and Eriksson, 1972) for the presence of PAS-positive, diastase-resistant globules in the liver of patients with HCC, a prevalence of about 10% was found. Both studies were conducted in populations in which this tumour is rare. Just how important AAT deficiency is as a cause of HCC in those parts of the world where the tumour is common has not yet been established. We have measured serum AAT concentrations by trypsin inhibitory capacity (STIC) or radial immunodiffusion (RID), determined AAT

* Correspondence and reprint requests to: Dr M. C. Kew, Department of Medicine, Witwatersrand University Medical School, Hospital Hill, Johannesburg 2001, South Africa.

‡ AAT subtypes are designated by letters according to their electrophoretic mobility, as F (fast), M (medium), S (slow) and Z (ultra slow). For details, see Fagerhol (1976).

phenotypes by acid-starch electrophoresis, and looked for PAS-positive diastase-resistant globules in the livers and tumours of a series of southern African blacks with HCC.

Seventy-seven unselected adult black patients with histologically proven HCC were studied. All but 4 of the patients were males. Blood was taken at the time of diagnosis and before treatment was begun. STIC was measured by the benzoyl-arginine-p-nitroanilide method of Erlanger *et al.* (1961) in 60 patients and serum AAT concentrations by RID (Schulman, 1973) in the remaining 17 patients. AAT phenotyping was determined in all patients by acid-starch electrophoresis as described by Fagerhol (1968). Non-tumorous liver tissue (normal or cirrhotic) and tumour tissue obtained from each patient either by percutaneous biopsy or at laparotomy or necropsy were stained with PAS after treatment with diastase and counterstained with haematoxylin. All the slides were prepared by one technician in a single session. A section from the liver of a patient with proven AAT deficiency and known to have PAS-positive diastase-resistant globules in the hepatocytes was included with the other slides as a control. The sections were then examined for PAS-positive inclusion globules. In patients with HCC and AAT deficiency, PAS-positive diastase-resistant globules are found in the malignant hepatocytes as well as in the non-tumorous liver tissue (Lieberman *et al.*, 1975). The non-tumorous liver tissue is cirrhotic in about 60% of our patients with HCC.

Control values for serum AAT concentrations by RID, and AAT phenotyping, were established in 630 black male blood donors, while those for STIC were determined in 100 healthy young adult black males.

STIC values in 60 HCC patients ranged from 1·40 to 3·18 mg trypsin inhibited per ml of serum, with a mean of 2·77 (the normal range is 1·0 to 1·7 mg/ml serum). All but one patient had a concentration greater than 1·7 mg/ml serum. In the remaining 17 patients, AAT concentrations determined by RID ranged from 270 to 450 mg% with a mean of 386 (the normal range is 180 to 320 mg%). With one exception, the values were greater than 320 mg%.

No case of homozygous Z phenotype of AAT was found by acid-starch electrophoresis. The phenotype distribution was similar to that found in the controls, in whom were also no homozygous Z phenotypes. Although not encountered in this group of healthy blacks, the Z phenotype does occur in this population (Prinsloo and Turnbull, unpublished).

In none of the HCC patients were intracytoplasmic PAS-positive, diastase-resistant globules observed, either in non-tumorous liver tissue or in the tumour.

In a prospective study using chemical, electrophoretic and histological criteria, we could find no case of AAT deficiency in 77 unselected black patients with HCC. It is therefore highly unlikely that AAT deficiency plays a major role in the aetiology of this tumour, which occurs so commonly in southern African blacks. Our findings are at variance with those of Berg and Eriksson (1972) and Norkin and Campagna-Pinto (1968), the former studying European and the latter American patients. There are 2 possible explanations for this. Firstly, the PAS-positive globules in the livers of their patients may have consisted of some substance other than AAT in the cytoplasm of the hepatocytes (Dekker and Krause, 1973; Palmer *et al.*, 1974). To be certain that these globules contain AAT, it is necessary to use either a specific immunofluorescent technique on the tissues, using fluorescein-labelled anti-human AAT (Delellis *et al.*, 1972) or the immunoperoxidase method (Palmer and Wolfe, 1976). This was not done in the 2 studies cited. As both were retrospective analyses, neither chemical estimations of STIC or AAT concentrations by RID, nor AAT phenotyping, were carried out on the patients' serum to confirm AAT deficiency. The second possible reason for the discrepant results is that HCC may be multifactorial in aetiology and that the tumour has different causes in different parts of the world. AAT deficiency could be an occasional cause of the tumour in most parts of the world where HCC occurs sporadically, but it is not a numerically important cause in southern Africa, and possibly not elsewhere in Africa or in the Far East where this tumour is common.

As we did not perform crossed-electrophoresis in phenotyping our patients, it may be argued that heterozygote Z phenotypes might have been missed. The results of the chemical and histological studies make this unlikely. Lieberman and his coworkers (1975) have seen only one MZ patient with an STIC greater than 1·3 mg trypsin inhibited per ml of serum and this was found shortly after a partial hepatectomy had been performed. All but 2 of our patients had STIC or AAT concentrations by RID above the upper limit of normal, and it is doubtful that a MZ or even a FZ phenotype would have achieved these levels. PAS-positive diastase-resistant globules were present in all of Eriksson and Hagerstrand's large series of patients with AAT deficiency (Eriksson and Hagerstrand, 1974). These globules were not demonstrable in the 2 patients in the present series with normal serum AAT concentrations or, indeed, in any of the patients.

With 2 exceptions, our patients had high AAT levels. AAT concentrations are known to rise in the blood during the course of malignant disease (Clark *et al.*, 1948). It has been suggested that the increase in AAT is an attempt by the body to defend itself against the spread and progression of cancer (Goetz *et al.*, 1972; O'Neill, 1974). Were this so, individuals with AAT deficiency might be expected to have an increased susceptibility to all forms of malignancy, and not only to HCC. Alternatively, high AAT values might simply reflect a non-specific acute-phase response to tissue injury or inflammation.

The authors acknowledge the support of the National Cancer Association of South Africa. The STIC estimations were carried out by the Analytical Division of the Department of Biochemistry of the National Research Institute for Occupational Diseases.

### REFERENCES

AAGENAES, O., FAGERHOL, M., ELGJO, K., MUNTHE, E. & HOVIG, T. (1974) Pathology and Pathogenesis of Liver Disease in Alpha$_1$-antitrypsin Deficient Individuals. *Postgrad. med. J.*, **50**, 365.

BERG, N. O. & ERIKSSON, S. (1972) Liver Disease in Adults with Alpha$_1$-antitrypsin Deficiency. *New Engl. J. Med.*, **287**, 1264.

CLARK, G. D. C., CLIFTON, E. E. & NEWTON, B. L. (1948) Antiproteolytic Activity of Human Serum with Particular Reference to its Changes in the Presence of and Considerations of its Use for Detection of Malignant Neoplasms. *Proc. Soc. exp. Biol. Med.*, **69**, 276.

DEKKER, A. & KRAUSE, J. R. (1973) Hyaline Globules in Human Neoplasms. *Arch. Path.*, **95**, 178.

DELELLIS, R. A., BALOGH, K., MERK, F. B. & CHIRIFE, A. M. (1972) Distinctive Hepatic Cell Globules in Adult Alpha$_1$-antitrypsin Deficiency. *Arch. Path.*, **94**, 308.

ERIKSSON, S. & HAGERSTRAND, I. (1974) Cirrhosis and Malignant Hepatoma in Alpha$_1$-antitrypsin Deficiency. *Acta med. scand.*, **195**, 451.

ERIKSSON, S. & LARSSON, C. (1975) Purification and Partial Characterisation of PAS-positive Inclusion Bodies from the Liver in Alpha$_1$-antitrypsin Deficiency. *New Engl. J. Med.*, **292**, 176.

ERLANGER, B. F., KOKOWSKY, N. & COHEN, W. (1961) The Preparation and Properties of Two New Chromogenic Substrates of Trypsin. *Archs. Biochem. Biophys.*, **95**, 271.

FAGERHOL, M. K. (1968) The Pi-system. Genetic Variance of Serum Alpha$_1$-antitrypsin. *Series Haematologica*, **1**, 153.

FAGERHOL, M. K. (1976) The Genetics of Alpha$_1$-antitrypsin and its Implications. *Postgrad. med. J.*, **52**, Suppl. 2, 73.

FELDMAN, G., BIGNON, J. & CHANINIAN, P. (1974) The Liver in Alpha$_1$-antitrypsin Deficiency. *Digestion*, **10**, 162.

GANROT, P. O., LAURELL, C. B. & ERIKSSON, S. (1967) Obstructive Lung Disease and Trypsin Inhibitors in Alpha$_1$-antitrypsin Deficiency. *Scand. J. clin. Lab. Invest.*, **19**, 204.

GOETZ, I. E., WEINSTEIN, C. & ROBERTS, E. (1972) Effects of Protease Inhibitors on Growth of Hamster Cells in Culture. *Cancer Res.*, **32**, 2469.

LIEBERMAN, J. (1974) Emphysema, Cirrhosis and Hepatoma with Alpha$_1$-antitrypsin Deficiency. *Ann. intern. Med.*, **81**, 850.

LIEBERMAN, J., SILTON, R. M., AGLIOZZO, C. M. & MCMAHON, J. (1975) Hepatocellular Carcinoma and Intermediate Alpha$_1$-antitrypsin Deficiency. (MZ Phenotype). *Am. J. clin. Path.*, **64**, 304.

NORKIN, S. A. & CAMPAGNA-PINTO, D. (1968) Cytoplasmic Hyaline Inclusions in Hepatoma. *Arch. Path.*, **86**, 25.

O'NEILL, F. J. (1974) Limitation of Nuclear Division by Protease Inhibitors in Cytochalasin-B-treated Tumor Cells. *J. natn Cancer Inst.*, **52**, 653.

PALMER, P. E., DELELLIS, R. A. & WOLFE, H. S. (1974) Immunochemistry of Liver in Alpha$_1$-antitrypsin Deficiency. *Am. J. clin. Path.*, **100**, 232.

PALMER, P. E. & WOLFE, H. S. (1976) Alpha$_1$-antitrypsin Depositiou in Primary Hepatic Carcinomas. *Archs. Pathol. Lab. Med.*, **100**, 232.

RAWLINGS, W., MOSS, J., COOPER, H. S. & HAMILTON, S. R. (1974) Hepatocellular Carcinoma and Partial Deficiency of Alpha$_1$-antitrypsin (MZ). *Ann. intern. Med.*, **81**, 771.

SCHULMAN, G. (1973) Accuracy and Precision in Measurement of Human Serum Immunoglobulins G, A and M. *S. Afr. J. med. Sci.*, **38**, 61.

SHARP, H. L. (1971) Alpha$_1$-antitrypsin Deficiency. *Hosp. Practice*, **6**, 83.

SHARP, H. L. (1976) The Current Status of Alpha$_1$-antitrypsin, a Protease Inhibitor, in Gastrointestinal Disease. *Gastroenterology*, **70**, 611.

WILLIAMS, W. D. & FAJARDO, L. F. (1974) Alpha$_1$-antitrypsin Deficiency. A Hereditary Enigma. *Am. J. clin. Path.*, **61**, 311.

ZWI, S., HURWITZ, S. S., COHEN, C., PRINSLOO, I. & KAGAN, E. (1975) Alpha$_1$-antitrypsin Deficiency—an Association with Hepatic Malignancy. *S. Afr. med. J.*, **49**, 1887.


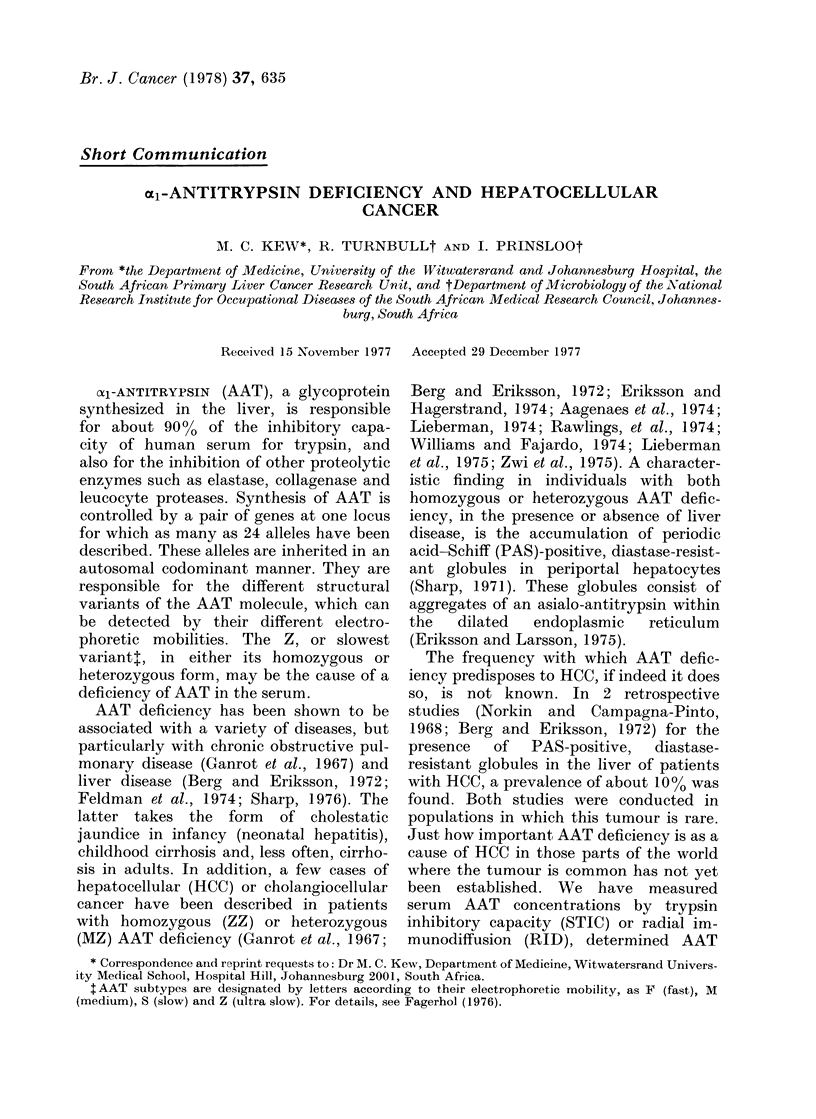

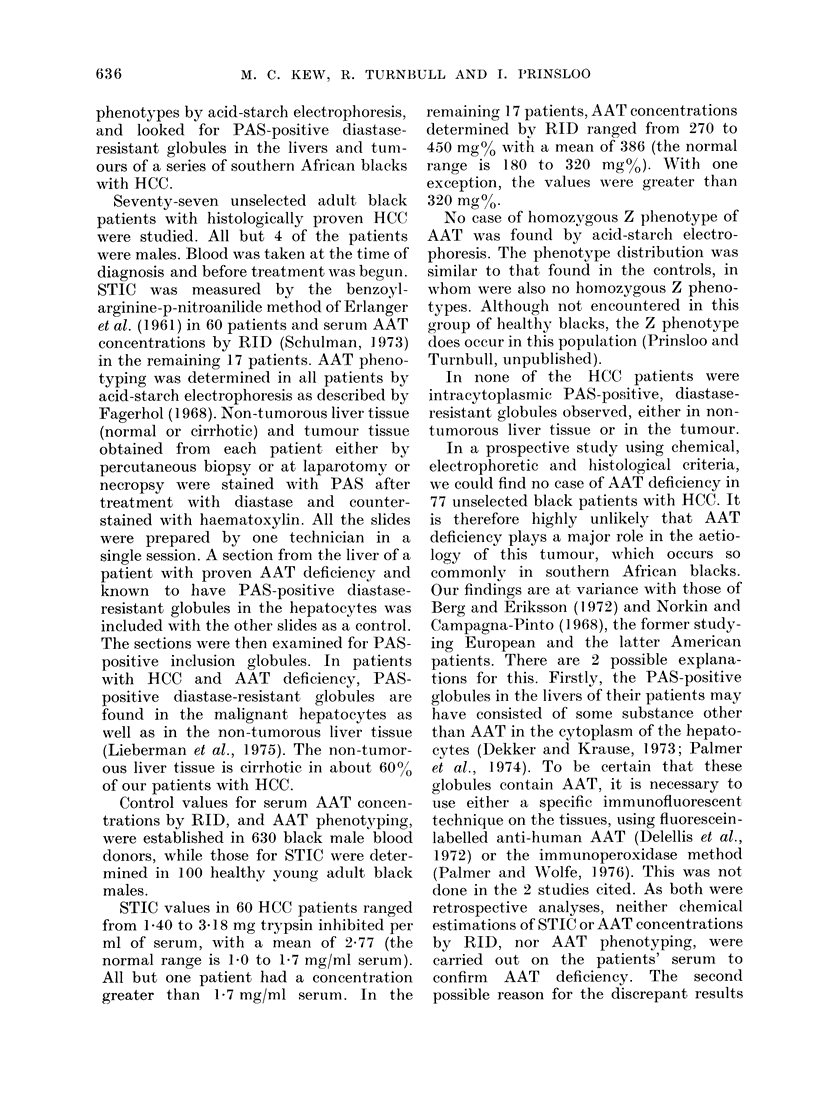

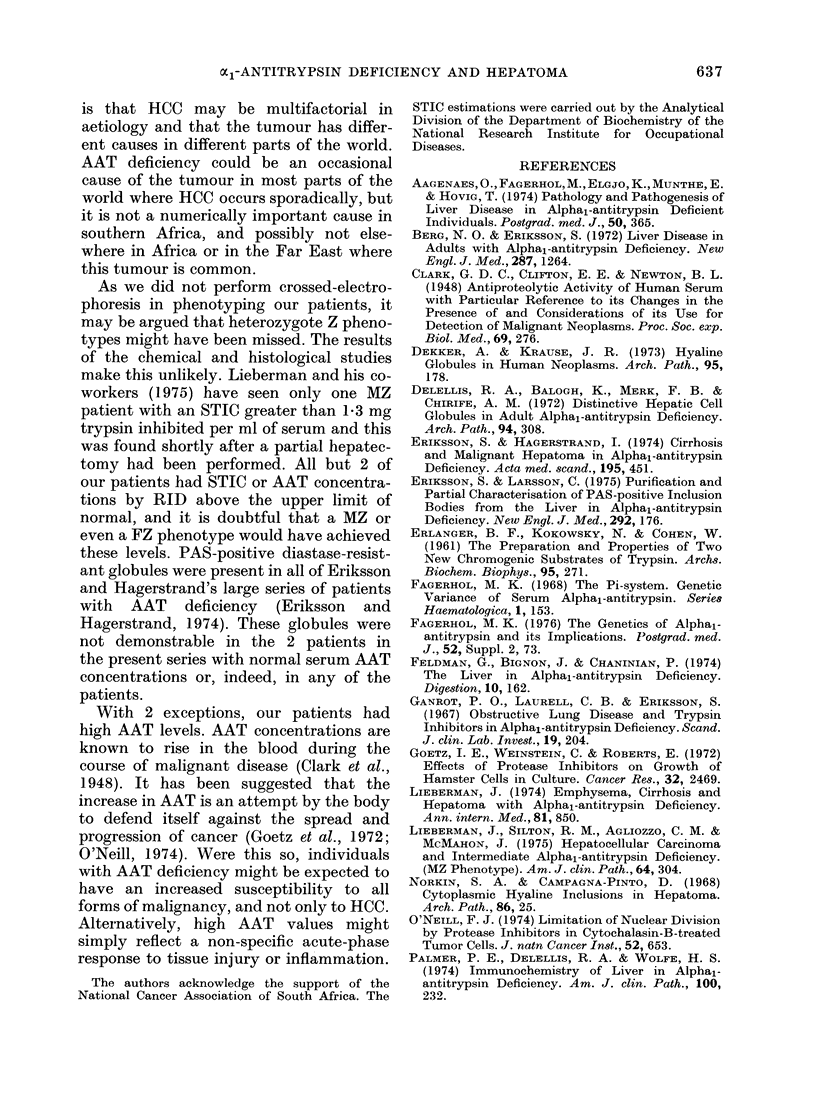

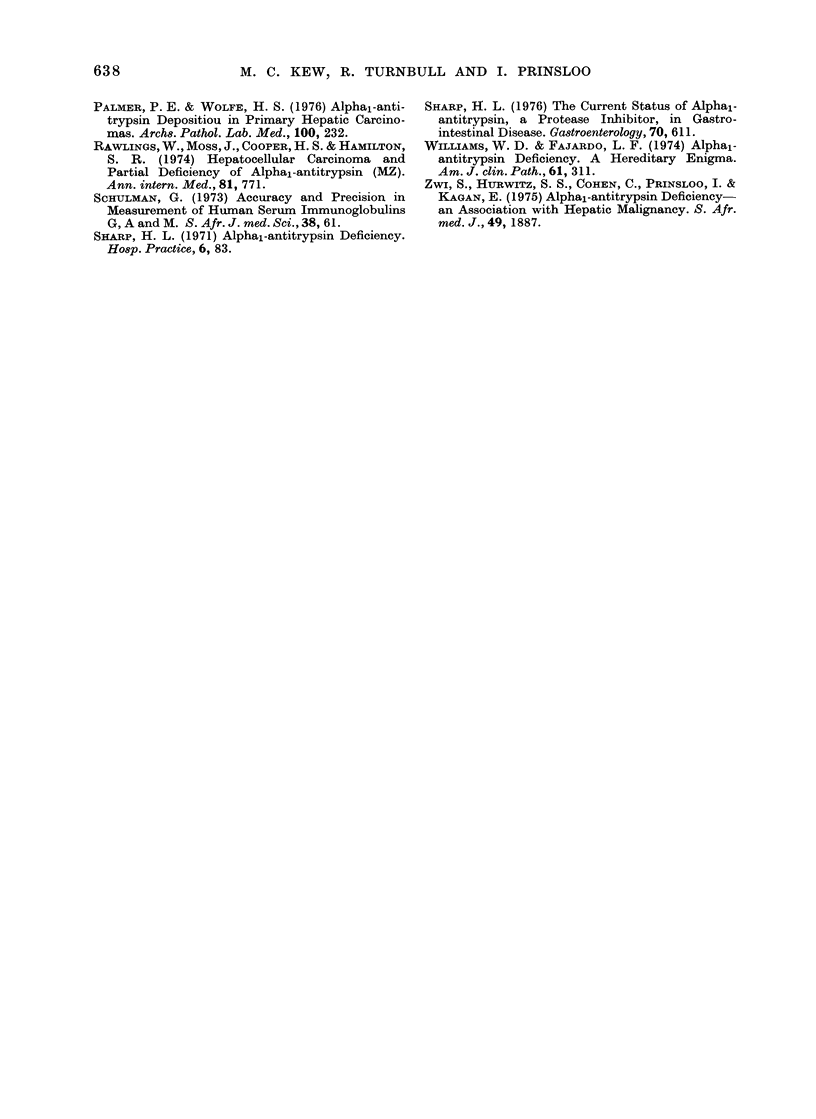


## References

[OCR_00274] Aagenaes O., TFagerhol M., Elgjo K., Munthe E., Hovig T. (1974). Pathology and pathogenesis of liver disease in alpha-1-antitrypsin deficient individuals.. Postgrad Med J.

[OCR_00280] Berg N. O., Eriksson S. (1972). Liver disease in adults with alpha-1 -antitrypsin deficiency.. N Engl J Med.

[OCR_00298] DeLellis R. A., Balogh K., Merk F. B., Chirife A. M. (1972). Distinctive hepatic cell globules in adult alpha-1-antitrypsin deficiency. A histochemical, immunohistochemical, and ultrastructural study.. Arch Pathol.

[OCR_00293] Dekker A., Krause J. R. (1973). Hyaline globules in human neoplasms. A report of three autopsy cases.. Arch Pathol.

[OCR_00315] ERLANGER B. F., KOKOWSKY N., COHEN W. (1961). The preparation and properties of two new chromogenic substrates of trypsin.. Arch Biochem Biophys.

[OCR_00304] Eriksson S., Hägerstrand I. (1974). Cirrhosis and malignant hepatoma in alpha 1-antitrypsin deficiency.. Acta Med Scand.

[OCR_00309] Eriksson S., Larsson C. (1975). Purification and partial characterization of pas-positive inclusion bodies from the liver in alpha 1-antitrypsin deficiency.. N Engl J Med.

[OCR_00331] Feldmann G., Bignon J., Chahinian P. (1974). The liver in alpha 1-antitrypsin deficiency.. Digestion.

[OCR_00342] Goetz I. E., Weinstein C., Roberts E. (1972). Effects of protease inhibitors on growth of hamster tumor cells in culture.. Cancer Res.

[OCR_00346] Lieberman J. (1974). Emphysema, cirrhosis, and hepatoma with alpha-1 antitrypsin deficiency.. Ann Intern Med.

[OCR_00351] Lieberman J., Silton R. M., Agliozzo C. M., McMahon J. (1975). Hepatocellular carcinoma and intermediate alpha1-antitrypsin deficiency (MZ phenotype).. Am J Clin Pathol.

[OCR_00357] Norkin S. A., Campagna-Pinto D. (1968). Cytoplasmic hyaline inclusions in hepatoma. Histochemical study.. Arch Pathol.

[OCR_00362] O'Neill F. J. (1974). Limitation of nuclear division by protease inhibitors in cytochalasin-B-treated tumor cells.. J Natl Cancer Inst.

[OCR_00375] Palmer P. E., Wolfe H. J. (1976). Alpha-antitrypsin deposition in primary hepatic carcinomas.. Arch Pathol Lab Med.

[OCR_00380] Rawlings W., Moss J., Cooper H. S., Hamilton S. R. (1974). Hepatocellular carcinoma and partial deficiency of alpha-1 antitrypsin (MZ).. Ann Intern Med.

[OCR_00395] Sharp H. L. (1976). The current status of alpha-1-antityrpsin, a protease inhibitor, in gastrointestinal disease.. Gastroenterology.

[OCR_00386] Shulman G. (1973). Accuracy and precision in measurement of human serum immunoglobulins G, A and M.. S Afr J Med Sci.

[OCR_00405] Zwi S., Hurwitz S. S., Cohen C., Prinsloo I., Kagan E. (1975). Alpha1-antitrypsin deficiency--an association with hepatic malignancy.. S Afr Med J.

